# Nontraumatic intra-diploic arachnoid cyst communicating with sphenoid bone and in close proximity to cavernous sinus in a known case of Wilson disease: A rare entity

**DOI:** 10.1016/j.radcr.2024.07.141

**Published:** 2024-08-21

**Authors:** Anshul Sood, Gaurav Vedprakash Mishra, Pratapsingh Parihar, Shreya Khandelwal, Nishtha Manuja

**Affiliations:** a Department of Radiodiagnosis, Jawaharlal Nehru Medical College, Datta Meghe Institute of Higher Education and Research, Sawangi (Meghe), Wardha, Maharashtra 442001, India; b Department of Medicine, Jawaharlal Nehru Medical College, Datta Meghe Institute of Higher Education and Research, Sawangi (Meghe), Wardha, Maharashtra 442001, India

**Keywords:** Nontraumatic, Intradiploic arachnoid cyst, Sphenoid bone, Cavernous sinus, Wilson disease

## Abstract

Arachnoid cysts can be intra-cranial or along the spinal cord. Intracranial arachnoid cyst is a very rare finding, trauma being the leading case of it. It is extremely rare for the development of intra-diploic arachnoid cyst without a traumatic history. We present a case of an intra-diploic arachnoid cyst communicating with the greater wing of the sphenoid and in close proximity to the cavernous sinus in a known case of Wilson disease for 22 years. Due to its low incidence, there is a gap in the knowledge and discussion of this cystic lesion, its pathophysiology and management, which are discussed in this case report.

## Introduction

The benign anomalies involving the intracranial arachnoidal membranes include arachnoid cysts, which are defined as a cerebrospinal fluid (CSF) filled collection. Very rarely, these cysts may appear in the intra-diploic spaces and are classified into traumatic and nontraumatic. Intra-diploic arachnoid cysts have a variety of nomenclature, including intra-osseous leptomeningeal cyst, intra-diploic CSF fistula, posttraumatic and traumatic arachnoid cysts [[1], [2], [3]]. The differential diagnosis of the arachnoid cyst is an epidermoid cyst, which can be differentiated based on the diffusion-weighted imaging sequence, with the latter showing diffusion restriction. We present a case of the rare appearance of an intra-diploic arachnoid cyst near the cavernous sinus in a known case of Wilson's disease.

## Case report

A 28-year-old male with a known case of Wilson disease, diagnosed 22 years ago and on medications since then, presented to the medicine outpatient department with complaints of weakness in all 4 limbs for the past 4 days, along with speech difficulties. The patient has had a history of seizures for the past 2 months, which are on and off, and the last episode of seizure was 7 days ago. The patient's sister was also diagnosed with Wilson disease, and she succumbed to it. Blood investigations were done, which revealed increased 24-hour urinary copper and spot urinary copper and low serum ceruloplasmin with associated proteinuria. On physical examination, Keyser-Fleischer rings and signs of upper motor neuron lesion were present, and the patient was advised of an MRI. The patient did not undergo any previous radiological investigations.

**Fig. 1:**
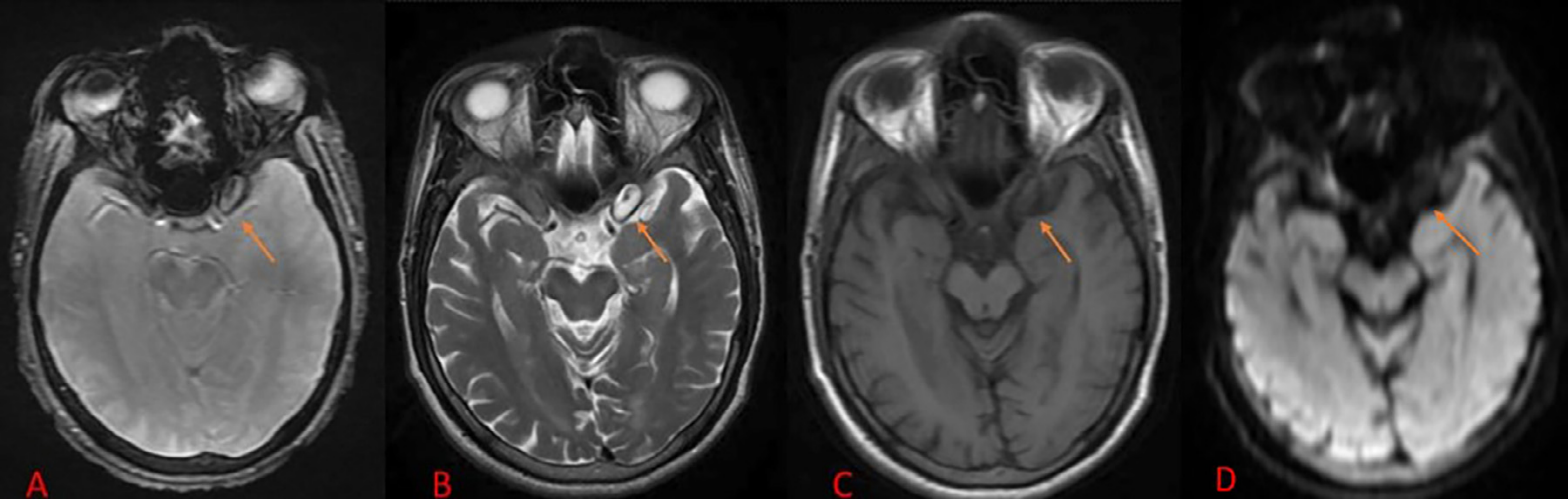
MRI brain axial images of Fluid attenuation inversion recovery sequence (A), T2 weighted imaging (B), T1 weighted imaging (C), and Diffusion Weighted Imaging (D) showing an intra-diploic lesion measuring approximately 14 × 7 mm adjacent to the left cavernous sinus, which appears isointense to CSF on T2WI, hypo to isointense to CSF on T1WI/FLAIR, and no restricted diffusion on DWI.

**Fig. 2:**
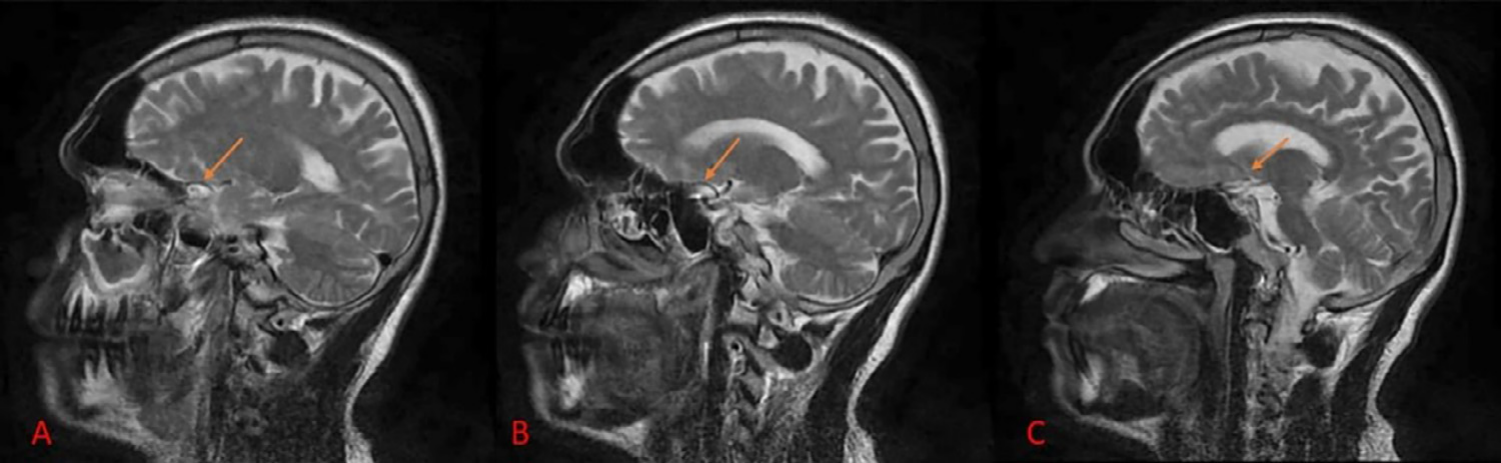
MRI brain sagittal image T2 weighted imaging (A-C) showing an intra-diploic lesion measuring approximately 14 × 7 mm adjacent to the left cavernous sinus, which appears isointense to CSF.

**Fig. 3:**
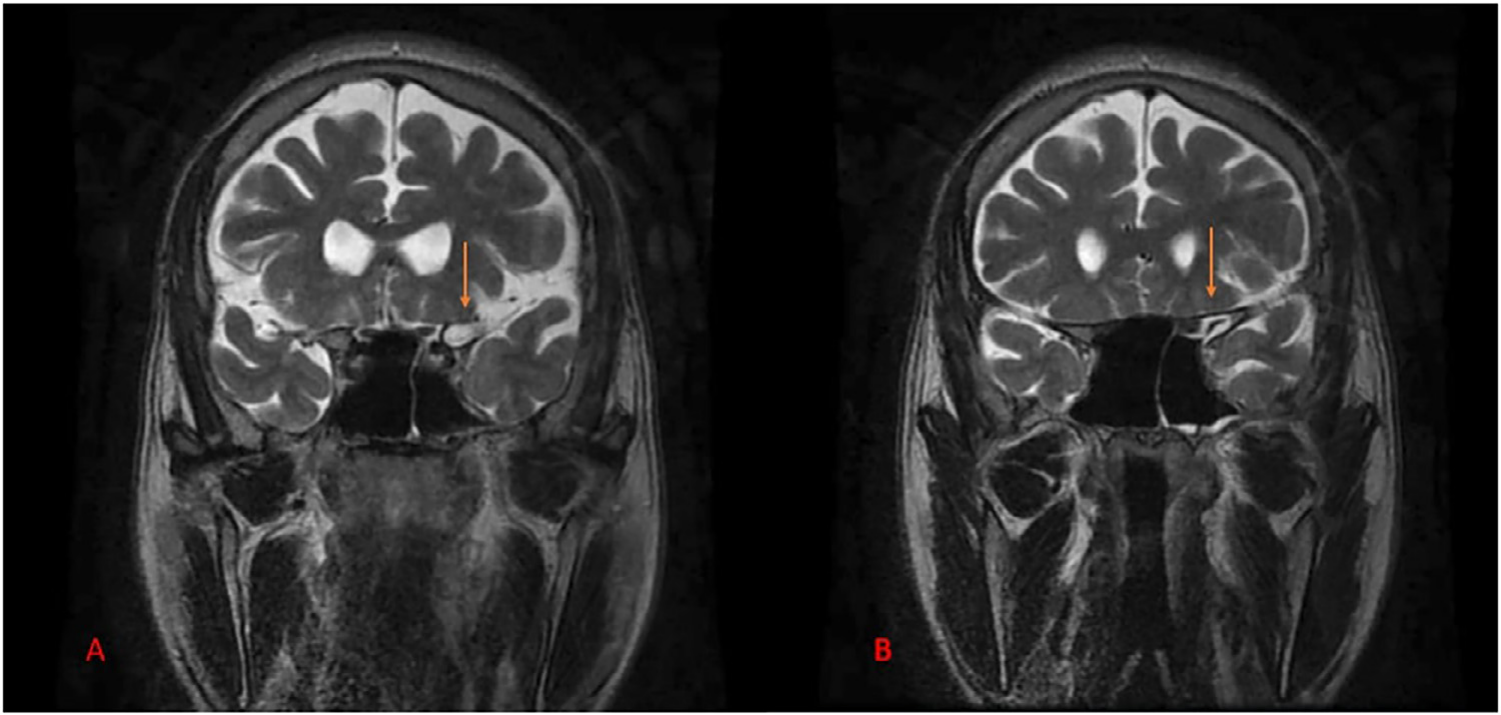
MRI brain coronal image T2 weighted imaging (A) and (B) showing an intra-diploic lesion measuring approximately 14 × 7 mm adjacent to the left cavernous sinus, which appears isointense to CSF and is noted communicating with the greater wing of the left sphenoid bone.

On MRI, an intra-diploic lesion measuring approx. About 14 × 7 mm was seen adjacent to the cavernous sinus on the left side, which appears isointense to CSF on T2WI, hypo to isointense to CSF on T1WI/FLAIR, showing no restriction on DWI and corresponding high signal in ADC, and no blooming noted at GRE sequences. The above features suggested the lesion to be an arachnoid cyst, which seems to communicate with the greater wing of the left sphenoid bone, as shown in Fig. 1, Fig. 2, Fig. 3.

## Discussion

Arachnoid cysts are benign and appear as cerebrospinal fluid (CSF) filled cystic structures [4,5]. These cysts are most seen in the middle cranial fossa and may be symptomatic [6]. They are mostly formed because of traumatic insult to the head, the occipital region being the most common location [[7], [8], [9]], and rarely are they present without a traumatic history. Intra-diploic arachnoid cyst is a very rare entity and may remain asymptomatic for a very long time. These cysts may cause diagnostic challenges, and radiological investigations like computed tomography and magnetic resonance imaging are a great support. To avoid any misinterpretation in diagnosing it, radiologic and clinicopathologic knowledge about the disease is important.

Arachnoid cysts are CSF filled cystic structures within the arachnoid layer, lined by collagen and arachnoidal cells. Its pathogenesis is still not completely clear due to its extremely rare incidence. They can be present at birth due to some congenital malformation in the early gestational life and are termed primary. These malformations can be caused due to abnormality in the drainage of CSF due to venous dysplasia or agenesis. These cysts may later grow due to secretion by the cells lining the cyst, unidirectional action of the valves or movement of liquid secondary to the vein pulsations [5]. There is no association of the arachnoid cyst with any disease in particular and has not been described in the recent literature. Its association with the Wilson disease has not been previously described in the recent literature. Secondary arachnoid cysts develop mostly due to trauma.

Traumatic insult to the head may cause bony defects and fractures. In these bony defects, arachnoid villi may extend, forming an intra-osseous cyst [9,10]. Leptomeningeal cysts and CSF fistula are other synonyms for post-traumatic arachnoid cysts [[10], [11], [12]]. Nontraumatic intra-diploic cysts are very rare and mostly are seen in the occipital region and rarely in temporal or frontal regions [8,10,11].

The size and location of the cyst determine the severity of the signs and symptoms. Symptoms may include bulging of the head, raised intracranial pressure, neurological defects, seizures, endocrine disorders, and psychomotor distortion.

It is important to rule out various differentials for the arachnoid cyst for which MRI plays a major role. As in the case of the epidermoid cyst, the cystic lesion appears isointense to CSF in both T1WI AND T2WI but hyperintense to CSF on FLAIR and shows restriction on DWI [13].

Neuroglial cyst follows CSF signal intensity on all sequences, as in the case of arachnoid cysts, but these are present in the white matter of the brain [14].

Other differentials may include subdural hygroma, which may appear isointense to CSF on T1WI and T2WI but hyperintense to CSF on the FLAIR sequence. Hemangioblastoma, pilocytic astrocytoma, etc., might mimic arachnoid cysts [15].

Asymptomatic arachnoid cysts do not need any management. Once the symptoms start to develop, these cysts must be excavated by a surgical approach, which includes marsupialization or fenestration through endoscopic or microscopic techniques or excision of the cyst [5].

Arachnoid cysts may be due to traumatic and nontraumatic causes. In the presented case, the arachnoid cyst is present at a very rare location and in close proximity to the cavernous sinus. Unusual location and low incidence may lead to misdiagnosis of the cyst. Proper clinical and radiological knowledge is of utmost importance in the diagnosis of the lesion and in differentiating it from other such lesions.

## Patient consent

An informed verbal and written consent were obtained from the patient.
